# A Current-Mode Analog Front-End for Capacitive Length Transducers in Pneumatic Muscle Actuators

**DOI:** 10.3390/mi15030377

**Published:** 2024-03-12

**Authors:** Guido Di Patrizio Stanchieri, Andrea De Marcellis, Marco Faccio, Elia Palange, Michele Gabrio Antonelli, Pierluigi Beomonte Zobel

**Affiliations:** 1 Electronic and Photonic Integrated Circuits and Systems Laboratory (EPICS Lab), Department of Information Engineering, Computer Science and Mathematics (DISIM), University of L’Aquila, Via Vetoio, Coppito, 67100 L’Aquila, Italy; andrea.demarcellis@univaq.it (A.D.M.); marco.faccio@univaq.it (M.F.); elia.palange@univaq.it (E.P.); 2 Industrial Automation and Mechatronics Laboratory (LIAM Lab), Department of Industrial and Information Engineering and Economics (DIIIE), University of L’Aquila, P.le Pontieri, Monteluco di Roio, 67100 L’Aquila, Italy; michelegabrioernesto.antonelli@univaq.it (M.G.A.); pierluigi.zobel@univaq.it (P.B.Z.)

**Keywords:** capacitive length transducers, current-mode analog front-ends, collaborative and soft robotics, pneumatic muscle actuators

## Abstract

This paper reports on the design, implementation, and characterization of a current-mode analog-front-end circuit for capacitance-to-voltage conversion that can be used in connection with a large variety of sensors and actuators in industrial and rehabilitation medicine applications. The circuit is composed by: (i) an oscillator generating a square wave signal whose frequency and pulse width is a function of the value of input capacitance; (ii) a passive low-pass filter that extracts the DC average component of the square wave signal; (iii) a DC-DC amplifier with variable gain ranging from 1 to 1000. The circuit has been designed in the current-mode approach by employing the second-generation current conveyor circuit, and has been implemented by using commercial discrete components as the basic blocks. The circuit allows for gain and sensitivity tunability, offset compensation and regulation, and the capability to manage various ranges of variations of the input capacitance. For a circuit gain of 1000, the measured circuit sensitivity is equal to 167.34 mV/pF with a resolution in terms of capacitance of 5 fF. The implemented circuit has been employed to measure the variations of the capacitance of a McKibben pneumatic muscle associated with the variations of its length that linearly depend on the circuit output voltage. Under step-to-step conditions of movement of the pneumatic muscle, the overall system sensitivity is equal to 70 mV/mm with a standard deviation error of the muscle length variation of 0.008 mm.

## 1. Introduction

Pneumatic muscles are actuators adopted for the fabrication of prosthetic devices, exoskeletons, and, in general, humanoids and robots for different application fields spanning from mechanical industry to physical and rehabilitation medicine [1,2,3,4,5,6,7]. In particular, the use of pneumatic muscles for collaborative robotics needs the highest levels of safety and reliability for applications designed to have frequent human–robot interactions. Thus, these applications require the real-time monitoring and control of the muscle pressure, length variations, and exerted force [6]. Among the pneumatic muscle architectures, McKibben muscles (MKMs) are unconventional pneumatic actuators fabricated by using hyper-elastic rubber tubes wrapped by a braided gauze [5]. The air pressure inside the MKM allows for the shortening of the muscle and the increase of its radial dimension. Two caps close the ends of the MKM tube and provide for mechanical and pneumatic seals. The MKM develops a force depending on the effective tube length and pressure values. Although some industrial applications utilize this kind of actuator, the compliance of the tube, in addition to the safety of the compressed air, suggests adopting it in rehabilitation medical applications, such as powered orthoses and prostheses [8]. For the patient’s safety and control of their interaction with these mechanical apparatus, force, length, and pressure sensors must be employed for the real-time control and actuation of the artificial muscles. In this sense, small force and pressure lightweight sensors are commercially available; in contrast, length sensors are typically bulky and not so easy to install in wearable devices [9,10,11,12,13]. However, several solutions have been reported in literature aimed to embed length sensors in pneumatic muscles [14,15,16,17]. As an example, linear variable displacement transducer sensors measure the pneumatic muscle length with high accuracy and good resolution, with the drawback that its extent is comparable with that one of the muscles. Additionally, the final system requires very accurate, fine mechanical fabrication to prevent mechanical backlashes and guarantee parallelism between the pneumatic muscle and the transducer axes, which are both difficult to be preserved for long muscle working periods [18,19]. Indirect real-time determination of pneumatic muscle length can be obtained by measuring the changes occurring to different muscle components; for example, this can be done by suitably positioning sensors around the muscle-braided gauze to infer the muscle length by measuring the variation of its tube diameter during the muscle elongation or contraction [14,20,21]. Nevertheless, electric and magnetic disturbances can affect the response of the sensors. This is particularly true when inductive and Hall-based sensors are employed [16,20,21]. Recently, a new version of MKM has been designed and fabricated with a structure embedding a cylindrical capacitor whose instantaneous capacitance has been experimentally demonstrated to be directly correlated to the actual muscle length [22,23]. The structure of this Capacitive-MKM (C-MKM) overcomes the drawbacks intrinsic of the abovementioned solutions since it: (i) directly measures the distance between the two ends of the muscle without the need of mechanical sensors; (ii) does not suffer from external electrical and magnetic disturbances; (iii) is easy to be mounted between the two muscle end caps; (iv) respects the compliance of MKM muscles. To take full advantage of the properties of the C-MKM, the present paper aims to describe the design, implementation, and characterization of a Capacitance-to-Voltage Analog-Front-End (CV-AFE) circuit capable of measuring the value of the C-MKM capacitance whose output voltage is directly proportional to the pneumatic muscle length during both its elongation and contraction phases in real-time [24,25,26,27,28,29,30,31,32,33,34,35,36,37,38,39]. In particular, CV-AFE circuits are employed in a large variety of medical [24,25,26,27,37,39] and industrial [28,29,30,31,32,33,34,35,36,37,38,39] applications. The integration of analog and digital electronic paradigms identifies the Capacitance-to-Digital Converters (CDC) that enable efficient digital data processing and data transmission [24,25,29]. On the other hand, when the specific application requests the output of the CV-AFE be a DC or AC voltage signals, the design architecture mainly employs a fully-analog approach; for example, this is the case for driving high-refresh-rate AMOLED displays [28], to control accelerometer, gyroscopes and positioning sensors [35,36,38]. The proposed CV-AFE circuit belongs to the latter class of converters since its DC output voltage can be employed to control the variations of the pneumatic muscle elongation or contraction with respect to a C-MKM initial value or rest condition by using suitable analog actuators [40,41,42,43,44]. The CV-AFE circuit has been implemented on a laboratory breadboard by using Commercial Off-The-Shelf (COTS) discrete components whose values have been chosen by considering variations of the C-MKM length up to 50 mm corresponding to a change of the C-MKM capacitance up to 25 pF. The design of the CV-AFE circuit allows it to vary its gain from 1 to 1000, so adjusting the circuit sensitivity and resolution depends on the specific application. A series of experimental measurements is reported and discussed for the complete electrical characterization of the CV-AFE circuit. As a case example of a real application, the capacitance of a fabricated C-MKM has been used as the input of the CV-AFE circuit to validate its capability to measure variations of pneumatic muscle length. The experimental findings prove that the CV-AFE circuit output voltage linearly depends on both the variations of the C-MKM capacitance and length. For a circuit gain equal to 1000, the maximum circuit sensitivity has been measured equal to 167.34 mV/pF, corresponding to about 70 mV/mm with a standard deviation error of the measured C-MKM length of 0.008 mm.

## 2. The CV-AFE Circuit for Capacitive Transducers: Main Parts and Operating Principles

**Figure 1:**
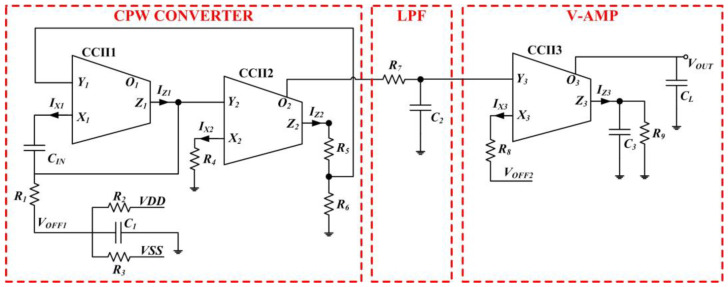
Schematic of the proposed CV-AFE circuit performing a capacitance-to-voltage conversion designed in a current-mode approach and implemented by employing COTS discrete components.

The proposed CV-AFE circuit is composed of three main blocks, as reported in Figure 1. The circuit converts the variation of an input capacitance *C_IN_* of any capacitive sensor/transducer into a DC voltage level *V_OUT_*. The solution has been designed in Current-Mode (CM) approach by employing the Second-Generation Current Conveyor (CCII) as the basic active blocks [45,46,47]. The CM approach simplifies the CCII circuitry once designed at transistor level with the advantages to operate at low voltage and low power with reduced Si area (i.e., reduced number of transistors). The main characteristics of the proposed architecture are the following: gain and sensitivity tunability, offset compensation and regulation, capability to manage different ranges of variations of the input capacitance *C_IN_*. Referring to Figure 1, the proposed CV-AFE circuit is composed by: (i) the Capacitance-to-Pulse-Width (CPW) CONVERTER block formed by CCII1, CCII2, *C*_1,_ *R*_1_, *R*_2,_ *R*_3,_ *R*_4_, *R*_5_, and *R*_6_ is an oscillator converting the value of the input capacitance *C_IN_* into a square wave voltage signal; (ii) a passive low-pass filer (LPF) is implemented by *R*_7_ and *C*_2_ to extract the mean value (i.e., the DC average component) of the output square wave signal generated by the CPW CONVERTER; (iii) a CM voltage amplifier (V-AMP) composed by CCII3, *R*_8_, *R*_9_, *C*_3_, and *C_L_*.

The main parameters of the square wave signal generated by the CPW CONVERTER depend on the value of the capacitance *C_IN_*. Therefore, a variation of *C_IN_* causes a change in the pulse width and of the frequency of the square wave signal. The value of the pulse width is extracted by the LPF and amplified by the V-AMP block whose voltage gain is set by the ratio *R*_9_/*R*_8_. Moreover, in Figure 1 in the CPW CONVERTER block, the resistive voltage divider composed of *R*_2_, *R*_3_ provides the voltage *V_OFF1_* and *C*_1_ stabilizes its DC level. In this way, it is possible to adjust the initial value of the square wave signal pulse width as a function of the initial value of the *C_IN_* for the specific application. In addition, the voltage *V_OFF2_* in the V-AMP block allows us to regulate/compensate the initial voltage offset, and the capacitors *C*_3_ and *C_L_* perform a further filtering operation of the output signal to achieve a more stable and less noisy DC voltage *V_OUT_* to increase the overall signal-to-noise ratio (SNR).

The voltages at the CCII *X* and *Y* nodes are given by the following equations:

(1) $$V_{X}=\frac{2R_{6}-R_{4}}{R_{5}+R_{6}}\;V_{DD}\;\;\;;\;\;\;V_{Y}=\frac{R_{4}}{R_{5}+R_{6}}\;V_{DD}\;$$

where $V_{DD}=-V_{SS}$ is the dual supply voltage that powers the circuit. Referring to Figure 1, the relationships between the voltages at the *X* and *Y* nodes of the CCII1 and CCII2 are equal to $V_{X}=\alpha V_{Y}$ and $V_{X}^{\prime }=\alpha^{\prime }V_{Y}^{\prime }$, respectively. Similarly, the relationships between the currents at the *Z* and *X* nodes of the CCII1 and CCII2 are equal to $I_{Z}=\beta I_{X}$ and $I_{Z}^{\prime }=\beta^{\prime }I_{X}^{\prime }$, respectively.

Consequently, the effective coefficient that considers both the CCII non-unitary gains and the circuit impedances is equal to:

(2) $$\gamma ={\alpha \alpha }^{\prime }\beta \beta^{\prime }\frac{R_{6}^{2}R_{5}}{{2R}_{4}\left(R_{5}+R_{6}\right)\left(\frac{R_{2}R_{3}}{R_{2}+R_{3}}+R_{1}\right)}$$

By performing the complete analysis of the response of the CPW CONVETER block, it is possible to determine the period, *T*, of the generated square wave pulse as a function of the variation of the input capacitance *C_IN_*:

(3) $$T=2R_{1}C_{IN}\left[ln\left(\frac{V_{X}}{V_{Y}+V_{OFF1}}\right)+ln\left(\frac{V_{X}}{V_{Y}-V_{OFF1}-\gamma V_{DD}\frac{C_{IN}}{C_{1}}}\right)\right]$$

The corresponding pulse width, PW, is equal to:

(4) $$PW=2R_{1}C_{IN}\frac{ln\left(\frac{V_{X}}{V_{Y}-V_{OFF1}-\gamma V_{DD}\frac{C_{IN}}{C_{1}}}\right)}{ln\left(\frac{V_{X}}{V_{Y}+V_{OFF1}}\right)+ln\left(\frac{V_{X}}{V_{Y}-V_{OFF1}-\gamma V_{DD}\frac{C_{IN}}{C_{1}}}\right)}$$

For the type of applications of interest of this paper, the variations of the input capacitance *C_IN_* are of the order of tens of picofarad. Under this assumption, it is possible to rewrite Equation (4) as:

(5) $$PW=2R_{1}C_{IN}\frac{ln\left(\frac{V_{X}}{V_{Y}-V_{OFF1}}\right)+\frac{\gamma V_{DD}C_{IN}}{C_{1}\left(V_{Y}-V_{OFF1}\right)}}{2ln\left(\frac{V_{X}}{V_{Y}-V_{OFF1}}\right)+\frac{\gamma V_{DD}C_{IN}}{C_{1}\left(V_{Y}-V_{OFF1}\right)}}$$

Under the approximations before stated, the generated square wave pulse width varies linearly with respect to the input capacitance *C_IN_*. The CPW CONVETER circuit can be modified as a function of the variation of the input capacitance needed for the specific application by suitably choosing the values of the resistances and of the capacitor *C*_1_.

## 3. The CV-AFE Circuit Implementation: Experimental Validation and Electrical Characterization

The proposed CV-AFE circuit powered by ±15 V dual supply voltage has been implemented on a laboratory breadboard by using COTS discrete components. For the electrical characterization of the circuit, the values of the input capacitance *C_IN_* have been considered to vary from 50 pF to 90 pF. These values of *C_IN_* are consistent with those achievable from the fabricated C-MKM (providing an elongation ranging about from 13 mm to 20 mm [22]) used in the input of the CV-AFE circuit for its characterization in the real application discussed below. Referring to Figure 1, the implemented circuit employs as CCII the AD844 by Analog Devices and the following values of the other components: *R*_1_ = 8.2 kΩ, *R*_2_ = 98.5 kΩ, *R*_3_ = 100 kΩ, *R*_4_ = 100 Ω, *R*_5_ = 10 kΩ, *R*_6_ = 1 kΩ, *R*_7_ = 2.2 kΩ, *R*_8_ = 330 Ω, *C*_2_ = 2.2 μF, and *C*_1_ = *C*_3_ = *C_L_* = 2.2 nF. In addition, by considering the CCII non-unitary gain parameters $\alpha =\alpha^{\prime }=0.92$ and $\beta =\beta^{\prime }=0.88$ of the AD844 components and the value of the coefficient γ from Equation (2), the effective *C_IN_* capacitance excitation voltage is equal to $\gamma V_{DD}=\pm 0.773\;V$.

The experimental electrical characterization of the CV-AFE circuit has been performed by using commercially available capacitors employed as *C_IN_* to validate the proposed solution for four values of the CV-AFE circuit V-AMP block voltage gains G = 1, G = 10, G = 100, and G = 1000. These voltage gains have been achieved by varying the ratio G = *R*_9_/*R*_8_ as follows: G = *R*_9_/*R*_8_ = (330 Ω/330 Ω) = 1, G = *R*_9_/*R*_8_ = (3.3 kΩ/330 Ω) = 10, G = *R*_9_/*R*_8_ = (33 kΩ/330 Ω) = 100, and G = *R*_9_/*R*_8_ = (330 kΩ/330 Ω) = 1000.

**Figure 2:**
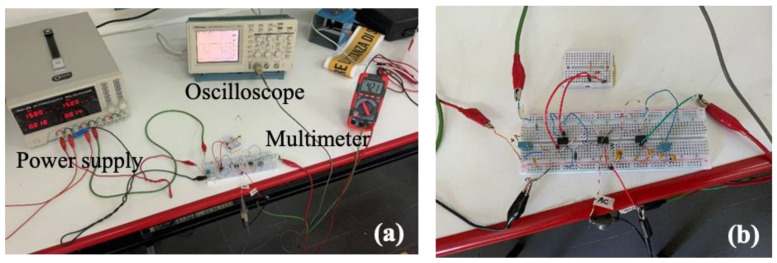
Experimental set-up for the electrical characterization of the CV-AFE circuit. Panel (**a**): overall view of the adopted instrumentation; Panel (**b**): detail of the implementation of the circuit on a laboratory board.

A picture of the experimental apparatus for the electrical characterization of the CV-AFE circuit is reported in Panel (a) of Figure 2 while Panel (b) shows the circuit implemented on a laboratory board. A three-channel RND 320-KA3305P laboratory electric power supply was adopted for the ±15 V dual supply voltage; a two-channel Tektronix TDS210 oscilloscope (bandwidth 0–60 MHz; sample rate 1 GS/s) was employed for measuring the frequency and pulse width of the square-wave signal; an RS-14 digital multimeter (accuracy ±1.0% of reading ±2 digits in the 1–200 V DC range) was used for measuring the output DC value.

**Figure 3:**
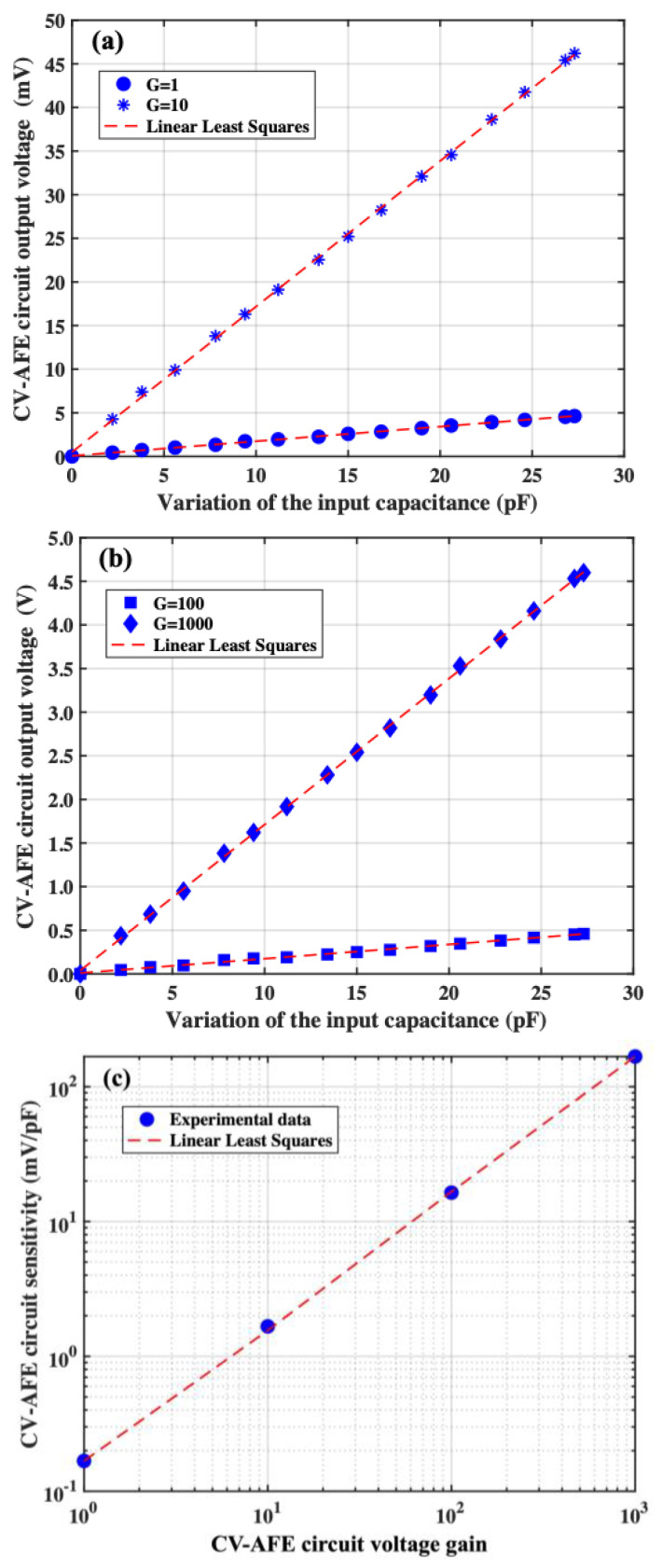
CV-AFE circuit output voltage as a function of the variation of input capacitance for different gains: Panel (**a**): G = 1 and G = 10; Panel (**b**): G = 100 and G = 1000; Panel (**c**): CF-AFE circuit sensitivity as a function of the circuit gain. The dashed red lines are the results of the linear least squares analysis of the experimental data.

In Panels (a) and (b) of Figure 3, the measured CV-AFE circuit output voltage as a function of the variation of the input capacitance *C_IN_* with respect to a starting value *C_min_* = 57 pF for all the previously set circuit gains are reported. At the beginning of each one of the measurements, the output voltage *V_OUT_* has been zeroed by acting on the value of the offset voltage *V_OFF2_* (see Figure 1). Moreover, as previously discussed, the value of the offset voltage *V_OFF1_* has been properly regulated to change the pulse width of the square wave signal generated by the CPW CONVERTER block, thus maximizing the CV-AFE circuit output voltage variations corresponding to the considered changes of the value of the input capacitance *C_IN_* (i.e., in the present case the maximum value of the input capacitance is *C_MAX_* = 84 pF) [22].

Referring to Panel (a) of Figure 3, the linear dependence of the CV-AFE circuit output voltage as a function of the variation of the input capacitance is proved by the linear least squares analysis of the experimental data that allows us to determine the circuit sensitivities with the corresponding standard deviations equal to S(G = 1) = (0.168 ± 0.001) mV/pF and S(G = 10) = (1.679 ± 0.008) mV/pF for the gain values equal to G = 1 and G = 10, respectively. The R-squared values for both the gain values are equal to R^2^ = 0.999 and the fractional uncertainties associated with the measured values of sensitivities are equal to ε(G = 1) = 0.6% and ε(G = 10) = 0.5%. By measuring the standard deviation of the CV-AFE circuit output voltage for 10 s acquisition time for each value of the input capacitance *C_IN_*, their average values have found to be equal to 0.2 mV and 0.33 mV for the gain G = 1 and G = 10, respectively. Therefore, the corresponding resolution in terms of capacitance achievable by the CV-AFE circuit are equal to r(G = 1) = 1.2 pF and r(G = 10) = 0.18 pF. Following the same procedure, the experimental results of the CV-AFE circuit output voltage for gains equal to G = 100 and G = 1000 are reported in Panel (b) of Figure 3, where the linear dependence of the CV-AFE circuit output voltage as a function of the input capacitance is proven by the linear least squares analysis of the experimental data.

The circuit sensitivities with the corresponding standard deviations are equal to S(G = 100) = (16.7 ± 0.2) mV/pF and S(G = 1000) = (167.3 ± 0.7) mV/pF for gains equal to G = 100 and G = 1000, respectively. The fractional uncertainties associated with these values of sensitivities are equal to ε(G = 100) = 0.8% and ε(G = 1000) = 0.4%, respectively. The corresponding R-squared values for both the gain values are equal to R^2^ = 0.999. Following the same procedure described above, for these values of the CV-AFE circuit gain, the average standard deviations of the CV-AFE circuit output voltage measured during 10 s acquisition time for each value of the input capacitance *C_IN_* have been found to be equal to 0.7 mV and 0.9 mV for the gain G = 100 and G = 1000, respectively. Therefore, the capacitance resolutions achievable by the CV-AFE circuit are equal to r(G = 100) = 42 fF and r(G = 1000) = 5 fF for gains G = 100 and G = 1000, respectively. The CV-AFE circuit sensitivity as a function of the circuit voltage amplification is reported in Panel (c) of Figure 3. In this case, the value of the slope calculated by the linear fitting procedure of the experimental data is equal to s = (0.167 ± 0.001) mV/pF, a value in very good agreement with respect to the experimental sensitivity S(G = 1) = (0.168 ± 0.001) mV/pF achieved for the circuit gain G = 1 (see Panel (a) of Figure 3). The experimental results reported in Figure 3 fully characterize the electrical features of the developed CV-AFE circuit that basically remain unaltered in terms of fractional uncertainties and resolutions for values of the circuit gain ranging from 1 to 1000.

## 4. The CV-AFE Circuit Application as Length Transducer in Pneumatic Muscle Actuators

**Figure 4:**
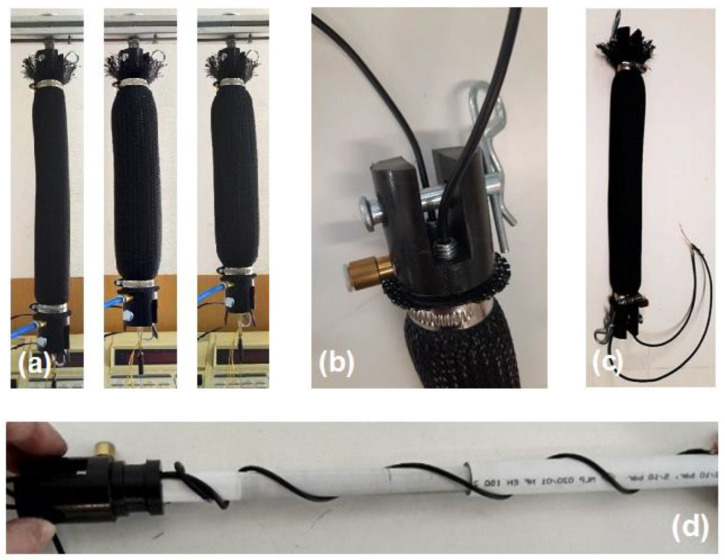
Panel (**a**): from left to right, three progressive compression phases of the developed C-MKM pneumatic muscle achieved by increasing its internal air pressure. Panel (**b**): detail of one C-MKM end with the air inlet/outlet port, the fork connection with the pin, and the couple of the shielded electrical cables. Panel (**c**): the C-MKM pneumatic muscle equipped with the external pins to measure the variation of its capacitance by means of the CV-AFE circuit. Panel (**d**): the assembly of the capacitive sensor to one of the C-MKM ends.

As a real case of application, the CV-AFE circuit implemented with COTS discrete components, as shown in Figure 3, has been employed to measure the change of the length of a fabricated C-MKM by using the value of the actual capacitance of the cylindrical capacitor embedded in the muscle as circuit input [22]. The C-MKM muscle is composed of an inner tube made of hyper-elastic rubber, an external braided gauze made of Polyamide 66, and the two ends. When air is inflated inside the inner tube, the deformation of the latter is guided by the outer gauze. Due to the inextensible threads of the gauze, the radial expansion of the tube causes its shortening, as shown in the Panel (a) of Figure 4. The C-MKM two ends provide to close the inner tube and assemble the two components of the embedded cylindrical capacitor. Moreover, one of the two ends provides for the inlet and outlet of the compressed air and for the passage of the shielded electrical cables, internally connected to the two components of the cylindrical capacitor. Both C-MKM ends are equipped with a fork connection, as shown in Panel (b) of Figure 4, to interface the pneumatic muscle to the external environment by two pins. Two metallic bands are placed around the ends for clamping the inner tube to the external gauze so ensuring pneumatic and mechanical seals. Panel (c) of Figure 4 shows the developed MKM prototype. The capacitive sensor is made of two concentric plastic multi-layer tubes. Each of them is made of an inner and outer polyethylene layer that embed an aluminum layer. The external diameters of the outer and inner tube of the capacitive sensor are equal to 26 mm and 20 mm, respectively. The aluminum layers of the two polyethylene layers are the capacitive foils of the prototyped cylindrical capacitor whose dielectrics are air and polyethylene. A metallic screw fixes a shielded cable at the external ends of both tubes. The external diameter, the overall length, and the active length of the C-MKM are equal to 36 mm, 380 mm, and 285 mm, respectively. Panel (d) of Figure 4 shows the assembly of the capacitive sensor to one of the C-MKM end.

**Figure 5:**
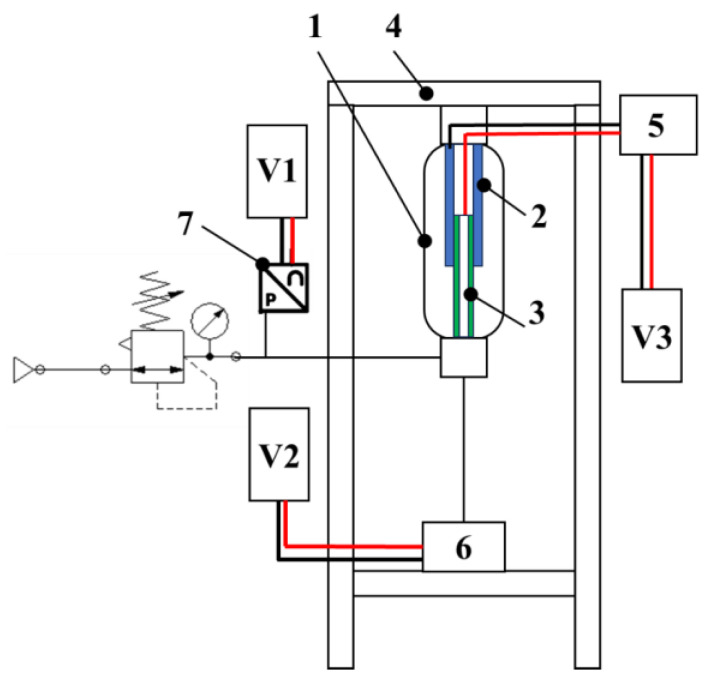
Block scheme of the experimental setup used for measuring the length change of the C-MKM using its capacitance as the input of the CV-AFE circuit: 1 is the C-MKM; 2 and 3 are the outer and the inner tube of the embedded capacitor, respectively; 4 is the portal testbench; 5 is the input of the CV-AFE circuit; 6 is the potentiometric transducer; 7 is the pressure transducer; V1, V2, and V3 are the voltmeters for the measurement of the output signals of the pressure transducer, the potentiometric transducer, and the CV-AFE circuit, respectively.

Figure 5 shows the schematic of the experimental setup that has been employed to measure the length change of the C-MKM using its capacitance as the input of the CV-AFE circuit and, for reference, a potentiometric transducer. In the schematic, 1 indicates the vertically mounted C-MKM, 2 and 3 indicate the outer and the inner tube of the C-MKM embedded capacitor, respectively, 4 indicates the portal testbench, 5 indicates the input of the CV-AFE circuit, 6 indicates the potentiometric transducer, and 7 indicates the pressure transducer. Three voltmeters have been used to measure the voltage output signals of the pressure transducer (V1), the potentiometric transducer (V2), and the CV-AFE circuit (V3). The potentiometric transducer (CELESCO DV301-0020-111-1110, f.s. 508 mm, output DC voltage range 0–10 V for 10 V DC power supply) is fixed on a portal testbench. The lower and upper C-MKM ends are joined by ball joints to the potentiometric transducer and to the test bench, respectively.

The output voltage of the potentiometric transducer allows us to determine the C-MKM length variations due to the change of its internal pressure. A MEMS pressure transducer (Honeywell ABPMANN004BGAA5, f.s. 4.0 bar, output DC voltage range 0.5–4.5 V for 5 V DC power supply) allows us to measure the pressure inside the C-MKM.

**Figure 6:**
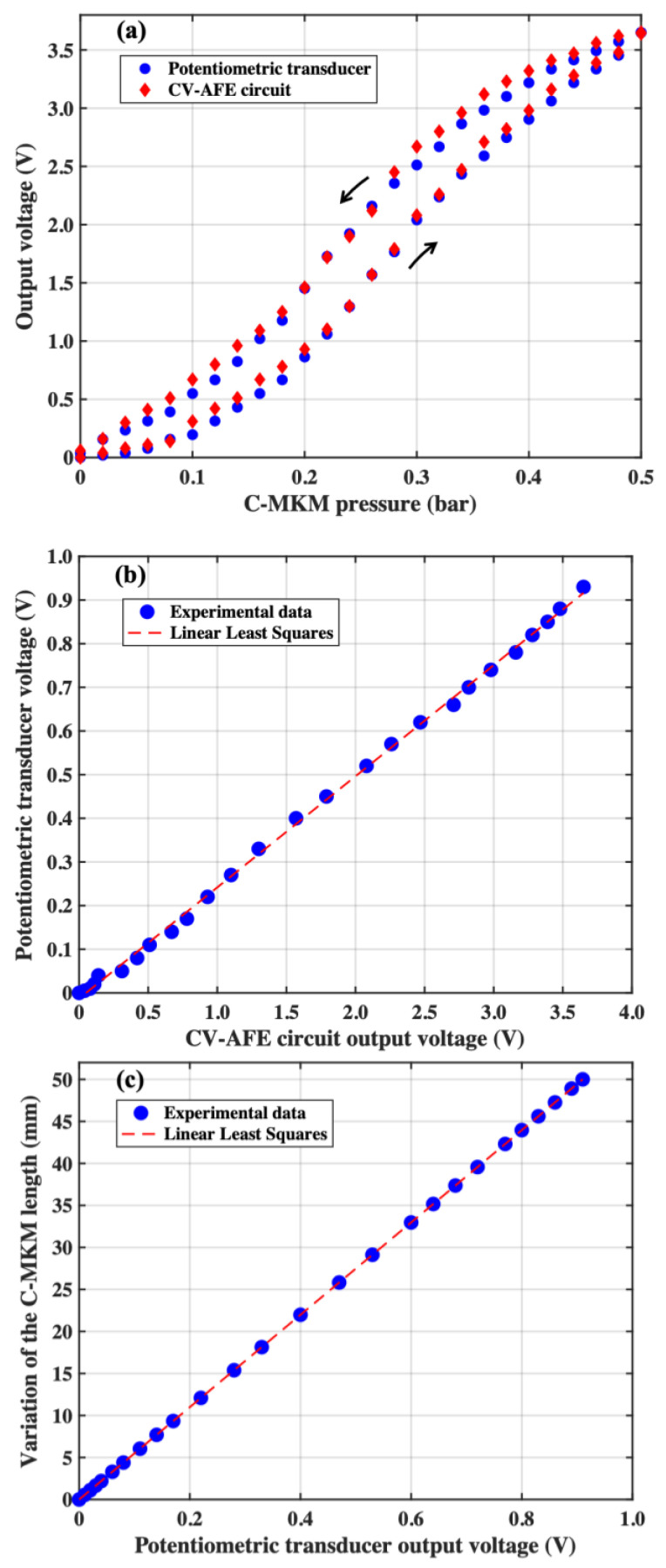
Panel (**a**): CV-AFE circuit and potentiometric transducer output voltages as a function of the C-MKM pressure. The lower and the upper arrows indicate the C-MKM elongation and shortening phases, respectively; Panel (**b**): potentiometric transducer output voltage as a function of the CV-AFE circuit output voltage; Panel (**c**): C-MKM length as a function of the potentiometric transducer output voltage. The dashed red lines are the results of the linear least squares analysis of the experimental data.

The CV-AFE circuit and the potentiometric transducer output voltages as a function of the C-MKM pressure are shown in Panel (a) of Figure 6. To better evaluate the experimental results, the potentiometric transducer output voltage has been normalized with respect to the corresponding CV-AFE circuit maximum output voltage setting the circuit amplification gain G = 1000. The normalization factor was found to be equal to 3.93. In Panel (b) of Figure 6, the linear relationship between the measured CV-AFE circuit output voltage for a gain G = 1000 and the potentiometric transducer output voltage during the C-MKM elongation phase is reported. Similar results are obtained during the C-MKM shortening phase. From the linear least squares analysis of the experimental data, the fitting equation $V_{pt}=\eta V_{AFE}$ holds with *η* = 0.254 ± 0.003 and a value of R^2^ = 0.98. It is worth noting that the inverse of *η* is equal to the above defined normalization factor.

As shown in panel (c) of Figure 6, the variation of the C-MKM length, *L*, with respect to a starting value is related to the potentiometric transducer output voltage *V_pt_* through the linear relation $L=\delta V_{pt}$. In the present case, assuming the C-MKM length is expressed in millimeter and the voltage *V_pt_* in Volt, the experimental measurements allow us to determine the proportionality factor equal to δ = (54.950 ± 0.003) mm/V.

**Figure 7:**
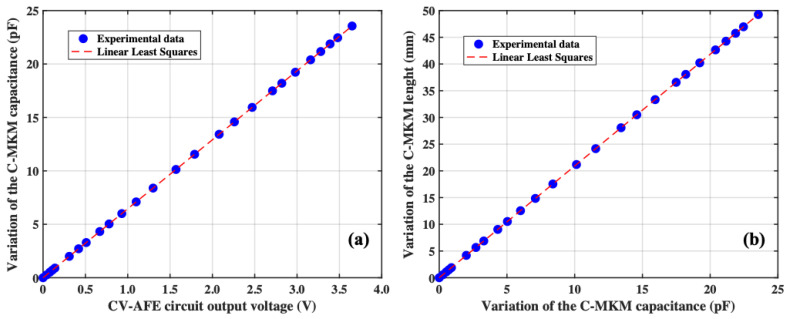
Panel (**a**): variation of the C-MKM capacitance as a function of the CV-AFE circuit output voltage setting a gain G = 1000; Panel (**b**): variation of the C-MKM length as a function of the C-MKM capacitance. The dashed red lines result from the linear least squares analysis of the experimental data.

Considering the linear dependence of the CV-AFE circuit output voltage with respect to the input capacitance *C_IN_* (see Figure 3 Panel (a) and Panel (b)), it is possible to determine the relationship between the variation of the C-MKM capacitance and the measured CV-AFE circuit output voltage as reported in Panel (a) of Figure 7. Based on this result, it is possible to establish the relationship between the variation of the C-MKM length and the variation of the C-MKM capacitance as shown in Panel (b) of Figure 7. For both the results reported in Figure 7, the linear dependence between the parameters reported there are demonstrated by the linear least squares procedures with R^2^ = 0.99.

**Figure 8:**
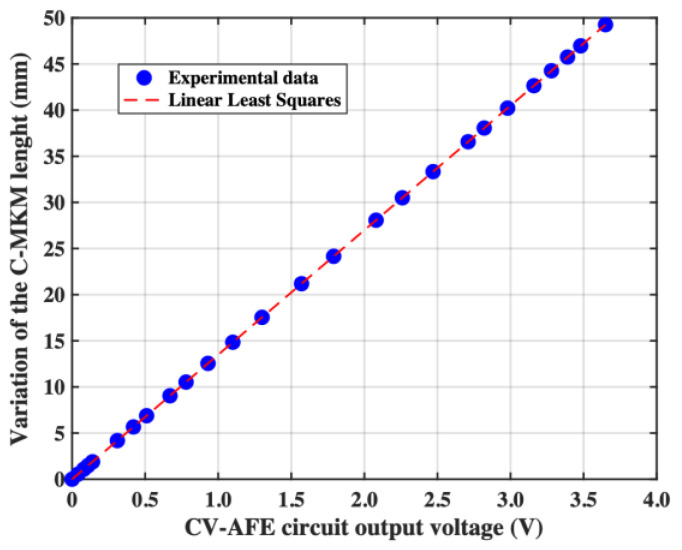
Variation of the C-MKM length as a function of the CV-AVE circuit output voltage. The dashed line results from the linear least squares analysis of the experimental data.

As a final step, it is possible to combine the results of Figure 7 Panel (a) and Panel (b) to obtain the dependence between the measured CV-AFE circuit output voltage and the variation of the C-MKM length as reported in Figure 8. Considering the results of the linear least square analysis of the experimental data in Panel (b) of Figure 3 for the CV-AFE circuit gain G = 1000, the data follow the relation $V_{OUT}=mC_{IN}+b$ with *m* = 167.3 mV/pF and *b* = 35 mV with the associated standard deviations SD(*m*) = 6 mV/pF and SD(*b*) = 10 mV. In the previous relation, *C_IN_* indicates the variation of the C-MKM capacitance. Considering the relationship between the variation of the C-MKM length and the potentiometric transducer output voltage $L=\delta V_{pt}$, it is possible find the equation describing the data of Figure 6:

(6) $$L=\delta \eta \left(mC_{IN}+b\right)$$

By using the statistical analysis, the standard deviation of the variation of the C-MKM length SD(*L*) can be found by fixing a value of *L* that, from Figure 8, corresponds to a specific value of the CV-AFE circuit output voltage *V_OUT_*. In the present case, the variation of the C-MKM length was set equal to the average value $\bar{L}=25.55\;mm$ that corresponds to ${\bar{V}}_{OUT}=1.83\;V$. This voltage is associated to a variation of the C-MKM capacitance $\bar{C}=11.6\;pF$.

Thus, Equation (6) can be rewritten as $\bar{L}=\delta \eta \left(m\bar{C}+b\right)$ and the standard deviation $SD\left(\bar{L}\right)$ can be found by applying the standard method of propagation of uncertainty considering that the parameters *η*, *m*, and *b* are mutually correlated (i.e., a variation of *m* changes the values of the other two parameters *η* and *b*, and vice versa). Assuming the relation between the standard deviations and the variances of these parameters, the resulting standard variation of $\bar{L}$ is $SD\left(\bar{L}\right)=7.7\;\mu m$. This value, achieved by experimental tests in static conditions, is considerably lower than the difference between two length values at the same pressure value in muscle shortening and lengthening tests, due to the hysteresis behavior of pneumatic muscles [48]. Considering the value of the slope obtained by the linear least squares analysis of the experimental data of Figure 8, the associated uncertainty of CV-AFE circuit output voltage circuit is equal to $r\left(V_{OUT}\right)=570\;\mu V$.

The main characteristics and performances of the proposed CV-AFE circuit compared with those ones of similar solutions in the literature are reported in Table 1. The reported implemented architecture achieves the best values in terms of sensitivity and resolution and allows us to adjust the baseline and the variation range of the input capacitance (i.e., detection range tunability) with the capability to also perform the offset regulation/compensation as a function of the specific application. Moreover, the CV-AFE circuit does not require the presence of any external signals and/or reference capacitors.

## 5. Conclusions

The paper reports on the design, implementation, and characterization of a current-mode analog-front-end circuit for capacitance-to-voltage conversion that can be used in connection with any capacitive sensor to drive actuators for applications in mechanical industry and rehabilitative medicine. The solution has been designed in the current-mode approach by employing the second-generation current conveyor as the basic blocks to convert the measured variation of capacitance into a corresponding value of a DC voltage signal. The circuit has been fabricated using commercial discrete components and the chosen architecture allows us to compensate and/or regulate the offset at the beginning of the measurements, to manage different ranges of variations of the input capacitance, and to vary its gain from 1 to 1000, thus achieving different values of sensitivity and resolution. From the electrical characterization, with the circuit gain equal to 1000, the measured circuit sensitivity has been found to be equal to 167.34 mV/pF with a resolution in terms of the variation of the input capacitance of 5 fF. As a real case of application, the capacitance of the capacitor embedded in a modified version of the McKibben pneumatic muscle has been used as the input for the front-end circuit. The reported experimental measurements demonstrate that the variation of the pneumatic muscle length depends linearly on the circuit output voltage. By setting the circuit gain equal to 1000, the system sensitivity is equal to 70 mV/mm with an associated standard deviation of the measured muscle length of about 0.008 mm. These experimental findings demonstrate that the proposed circuit architecture can be usefully employed with a large variety of capacitive sensors and actuators in collaborative robotics for industrial and medicine applications. The transistor-level design for the future fabrication of the integrated version (i.e., the ASIC) of the proposed CV-AFE circuit by a microelectronic solution in TSMC 180 nm standard CMOS Si technology is currently in progress. This will allow us to minimize the circuit size as well as to improve its overall performances and reduce the supply voltage and the power consumption.

## Figures and Tables

**Table 1:** Comparison of the main characteristics of the proposed CV-AFE circuit.

Parameters	[39]	[37]	[38]	[34]	This Work
Year	2015	2016	2017	2023	2024
Implementation type	Discrete components	Integrated	Discrete components	Integrated	Discrete components
COTS components	2 (LM555 + LM2917)	n/a	4 CCII (AD844)	n/a	3 CCII (AD844)
Sensitivity (mV/pF)	10	54	37.5	95	167.3
Gain/sensitivity tunability	no	yes	no	no	yes
Resolution (fF)	N/A	10	N/A	10.6	5
Capacitance variation range (pF)	0.01–800	0.01–27	140–260	0.01–24	57–84
Capacitance variation range tunability	no	no	yes	yes	yes
External signals	not required	not required	not required	required	not required
External reference capacitors	not required	not required	required	required	not required
Offset compensation	No	no	no	no	yes
Capacitive sensing applications	Body movement measurements	pH measurements	Position measurements	Dielectric measurements	Pneumatic muscle length measurements

## Data Availability

Data are contained within the article.
